# Effects of physical distancing by COVID-19 pandemic on diet quality, neurological and immunological markers, and fecal microbiota of Brazilian older women

**DOI:** 10.3389/fnut.2022.972100

**Published:** 2022-09-14

**Authors:** Priscila Larcher Longo, Rita de Cassia de Aquino, Sandra Regina Mota Ortiz, Roque Santos de Oliveira, Aline Gavioli, Jônatas Bussador do Amaral, Fernanda Rodrigues Monteiro, Raquel Riyuzo de Almeida Franco, Giovana Rebonatti Mereu, André Luis Lacerda Bachi, Alexandre José Bancher de Lima, Gilberto Candido Laurentino, Marta Ferreira Bastos

**Affiliations:** ^1^Postgraduate Program in Aging Sciences, São Judas Tadeu University, São Paulo, Brazil; ^2^ENT Laboratory, Department of Otorhinolaryngology, Federal University of São Paulo, São Paulo, Brazil; ^3^Postgraduate Program in Health Science, University of Santo Amaro, São Paulo, Brazil; ^4^Albert Einstein Israelite Hospital, São Paulo, Brazil

**Keywords:** aged, food intake, microbiome, inflammation, social distancing

## Abstract

Physical distancing was used to prevent transmission of COVID-19, however there are concerns that this may promote harmful impacts on health, such as reduced levels of physical practice and changes in food intake and gut microbiota composition. This study evaluated the impacts of 6 months physical distancing on Brazilian older women upon body mass index (BMI), strength, physical activity level (IPAQ), eating habits, neurological markers (brain-derived neurotrophic factor-BDNF and cortisol), cytokines (IL-2, IL-5, IL-6, IL-10, interferon-IFN-γ, tumor necrosis factor-TNF-α), aging-associated markers (vascular endothelial growth factor-VEGF, insulin-like growth factor-IGF-1, klotho and thymic stromal lymphopoietin-TSLP), besides specific groups of fecal microbiota. Fifteen women, over 60 years old, residents of São Paulo state (Brazil), were evaluated in March and in September 2020. The older adult women, with a mean age 66 ± 6.2 years presented significantly increased BMI and high effect size for non-protective foods consumption, reduced light physical activity and strength 6 months following the physical distancing. Furthermore, the serum concentration of IFN-γ, IGF-1, and IFN-γ/IL-5 were significantly higher, while lower concentration of IL-2 and IL-5 were observed 6 months after the physical distancing. Significant increase was noted only to *Blautia spp.* abundance after 6 months of physical distancing. Several correlations were observed at both before and after physical distancing, however, interestingly, many of them were lost or inverted 6 months following, while new ones emerged. Taken together, these results showed that lifestyle changes and stress conditions addressed by physical distancing from the COVID-19 pandemic impacted the health of older women included in the present study. Therefore, future follow-up studies are essential to propose interventions in order to restore the health conditions observed before the pandemic period, and thus to maintain the quality of life of older adults in different socioeconomic contexts.

## Introduction

COVID-19 is an acute respiratory illness caused by Severe Acute Respiratory Syndrome Coronavirus 2 (SARS-CoV-2), spread person-to-person through close contact (1, 2), presenting higher severity for older adults’ population (3). Although several policies have been employed to decrease transmissions, such as improving hygiene habits and mask-wearing, the physical distancing recommendations or lockdown was implemented as the best way to prevent contamination in most countries (4).

It has been recognized by literature that aging is a complex process involving morphological, physiological, biochemical, social, and psychological modifications that may be responsible for the increased risk of infections and comorbidities (5, 6). It is essential to emphasize that COVID-19 elderly deaths and physical enforced isolation could contribute to worse anxiety for self-identifying as “at risk” in this population.

The increased sedentary behavior and decreased physical activity during home confinement caused by COVID-19 pandemic has been shown. Ammar et al. (7) described that the number of minutes/week of vigorous intensity activities decreased by 33.1%, while moderate physical activities and walking were reduced by 33.4% and 34% respectively. Although some strategies to minimize the consequences of sedentary behavior have been proposed, the impacts of sedentarism during physical distancing in older adults deserve to be better investigated.

Regarding food intake, one of related factors more associated with diet quality of the older people in physical distancing was the difficulty to access fresh foods, especially fruits and vegetables, and greater consumption of ultra-processed foods or it’s purchased in establishments close to their homes, which favored the consumption of breads and processed meat. In addition, physical distancing impacts mental health and in the development of stress, anxiety, and depression, which in turn also influence food choices (8–10).

Social interaction and daily activities without restriction, as those experienced before COVID-19 pandemic, stimulate the musculoskeletal, cardiovascular, respiratory, and nervous systems (11). Therefore, physical activity generates benefits for physical health of older adults, stimulating muscle contraction, strength, energy expenditure, decreasing systemic inflammation and oxidative stress, reducing prevalence of chronic diseases, and geriatric syndromes such as sarcopenia and frailty (12), besides to benefiting gut microbiota composition (13). In addition, physical activity acts on brain health inducing positive changes as the enhancement of neurogenesis, angiogenesis, and synaptogenesis, caused by neurotrophins and growth factors, such as the brain-derived neurotrophic factor (BDNF) (14, 15).

Some authors (16–19) suggested that physical distancing for pandemic control decreases social connectedness and is associated with extensive hygiene habits, use of household disinfectants and cleaning products, travel barriers, and changes in habits which could significantly impact on the transfer of microorganisms within the population, and microbiome composition. This disruption in microbial sharing and inability for reinoculation (17) could be associated with loss of bacterial diversity causing dysbiosis with deleterious consequences for the host (16) impacting human health over the long term (17), mediating pathogen susceptibility and nosocomial infections (18).

Thus, it was hypothesized that physical distancing due COVID-19 pandemic that restricted mobility, leading to a recluse and sedentary behavior could impact the health of older adults repercussing a long term. Therefore, this longitudinal study aimed to verify the impacts of 6 months of physical distancing in Brazilian older women upon: eating habits, neurological (BDNF and cortisol), immunological [Interleukin (IL)-2, IL-5, IL-6, IL-10, interferon-gamma (IFN-γ), and tumor necrosis factor-alpha (TNF-α)] and aging-associated markers [vascular endothelial growth factor (VEGF), insulin-like growth factors 1 (IGF-1), and klotho and thymic stromal lymphopoietin (TSLP)], and fecal microbiota composition.

## Materials and methods

### Participants

The Research Ethics Committee of São Judas Tadeu University approved this longitudinal study (approval number 3.373.066). Initially, the older adults were invited, via flyers and social media (Facebook, Instagram, and WhatsApp), to participate in a specific physical protocol, part of extension project “Aging with strength” of the Postgraduate Program in Aging Science at São Judas Tadeu University in February 2020. In case of interest, the prospective participant received and signed the informed consent term. Older adults were included if their self-reported clinical conditions enabled them to do regular physical activity, and if there was no cognitive decline detected by researchers (Mini-mental score > 23 points). After evaluation by a specialist in geriatric medicine, older adults presenting unstable cardiovascular disease, acute infections, tumors, and knee or hip prostheses were excluded from the current study.

The initial analyses (named T0) were performed in March 2020, before physical distancing, which was instituted by São Paulo State government on March 22nd. This prevented launch of the initial extension project, that would have been carried out in person (physical training program). Thus, an alternative project was started instead, with weekly follow-up of all participants via phone calls and/or WhatsApp; none of them mentioned difficulties using smartphones or information and communication devices. Some older adults agreed to participate in the collection of new samples on 29 September 2020 (named T1), after 6 months of physical distancing. It is essential to point out that participants who reported positive tests for COVID-19 at any point were excluded from all analyses. A total of 35 older adults (male: *n* = 3, female: *n* = 32) were included in the study at T0, all of which were contacted for new assessments at T1. However, 19 participants were hesitant because of the pandemic (male: *n* = 3, female: *n* = 16), and one older woman was excluded due to a positive test for COVID-19. Thus, fifteen older women with no cognitive decline were included in this study, and all instruments and assessments were performed before the pandemic period (T0) and after 6 months of physical distancing (T1).

### Body mass index and physical activity assessment

Body mass index was calculated as body weight (kg) divided by squared height (m^2^). In order to assess body weight, a digital scale (Filizola, São Paulo, Brazil) with a 100 g precision was used; height was measured using a portable stadiometer with a 0.1 cm precision, and the results were expressed as kg/m^2^. The physical activity of participants was assessed though the International Physical Activity Questionnaire (IPAQ, short version) validated in Brazil by Matsudo et al. (20) and adapted for the older adult population. The IPAQ measures health-related physical activity and comprises items to assess the frequency and duration of physical activity in three ranges of intensity: vigorous/intense physical activity (8.0 metabolic equivalent [METs]), moderate physical activity (4.0 METs), and low/light physical activity (3.3 METs). The results were calculated in accordance with collected data, frequency, and duration of physical activity as well as estimated energy expenditure, and expressed in minutes per week (min/week).

### Strength test

Strength was assessed using a Jamar dynamometer (Lafayette Instrument Co., Lafayette, LA, United States), that measures the amount of grip strength when producing an isometric contraction of the hand muscles. Participants’ position followed the guidelines of the American Society of Hand Therapists (21). The testing protocol consisted of three repetitions of 5 s in maximal isometric contraction of the dominant hand, with a rest period of at least 60 s. The highest strength value between the three attempts was considered for analysis, with results shown in kilograms of force (Kgf).

### Food intake and diet quality

A screening instrument developed by Bailey et al. (22) with older people residents of Pennsylvania (United States), called DST (Dietary Screening Tool) was used to assess food intake and diet quality using dietary marker scores. With this questionnaire, it is possible to obtain a total score (up to 100 points) allowing characterization at three levels: presence of dietary risk, possible presence of dietary risk and absence of dietary risk. The DST is composed of twenty-six questions that assess the frequency of food groups consumption: (1) cereals and whole grains (15 points); (2) whole fruit and fruit juice (15 points); (3) vegetables (15 points); (4) milk and dairy products (10 points); (5) lean meats and processed meat (20 points); (6) fats, sugars and alcoholic beverages (25 points), with the consumption of some type of multivitamin and mineral supplement, adding 5 “extra” points. The answers were obtained by interview with a nutritionist, and the points for each participant were added up, representing the maximum scores related to food consumption, with values related to the highest or lowest intake frequency. This frequency regards how many times a food group was consumed, and it is established using the DST tool. Differences in consumption of some food groups (i.e., fruits, vegetables, and processed meat) were assessed separately.

The data collected were grouped and classified according to the evaluation of food intake frequency considered to be health-promoting (DST-protective foods) and non-health-promoting foods (DST-non-protective foods), based on the classification of the Food Guide for the Brazilian Population (23). Protective foods (markers of healthy eating) were represented by consumption of fruits, vegetables, and beans (*in natura* or minimally processed foods), and non-protective foods (markers of unhealthy eating) were represented by the consumption of processed meat, sweetened beverages, instant noodles, as well as consumption of sweets, candies, and sweet cookies (ultra-processed foods).

### Blood sampling and serum markers analysis

Peripheral whole blood (10 mL) was collected by venipuncture from the radio humeral venous plexus in vacutainer tubes without additives. The blood samples were centrifuged at 3.000 rpm for 10 min, and serum was collected, aliquoted, and stored at −80°C until assay. Serum concentrations of neurological (BDNF, cortisol), as well as aging-associated markers (VEGF, IGF-1, klotho, and TSLP) were analyzed by ELISA kits (R&D system, Minneapolis, MN, United States). Immunological markers (IL-2, IL-5, IL-6, IL-10, IFN-γ, and TNF-α) concentrations were determined by multiplex analyses using commercially available kits (Legendplex, Biolegend, San Diego, CA, United States) following the manufacturer’s instructions and recommendations.

### Fecal microbiota sampling and analysis

Samples were collected by the participants, who received instructions to freeze and transport their stool to the laboratory.

For fecal microbiota analysis, DNA extraction was performed with PowerSoil DNA Isolation Kit (MO BIO laboratories, Inc., Carlsbad, CA, United States). Amplification of hypervariable regions V3-V4 of the *16SrRNA* gene was performed using QIAseq 16S/ITS Region Panels (Qiagen) and sequencing was performed using MiSeq Reagent kit (Illumina, San Diego, CA, United States) at the Clinical Molecular Biology laboratory of Albert Einstein Israelite Hospital (São Paulo, Brazil).

Sequence data was analyzed using QIIME2 (Quantitative Insights into Microbial Ecology, v. 2021.8) software package. Raw fastq reads were denoised, merged and assessed for chimeras to produce amplicon sequence variants (ASV) using the DADA2 (Plugin version 2021.8.0) pipeline. Taxonomic classification was assigned using the SIL-VA database, specific for the V3/V4 *16SrRNA* region (version 138). All taxonomic classifications were implemented within QIIME2 and assigned using the plugin qiime feature-classifier classify-sklearn (24).

### Statistical analysis

The statistical analysis was performed using software (GraphPad Prism 9.0, GraphPad Software Inc., CA, United States). Initially, all data was examined for normality using the Kolmogorov Smirnov and Shapiro Wilk tests. In case no normality was achieved, non-parametric methods were used. Comparisons of serum marker concentrations and microbiota composition for T0 (before the pandemic period) and T1 (after 6 months of physical distancing) were performed using Wilcoxon, while BMI, physical activity level, strength, and food intake analyses were performed using the Student *t*-test. Furthermore, Cohen’s *d* was calculated to evaluate the effect size of different results using Cohen’s *d* = (*M*_2_−*M*_1_)/*SD*_*D*_, where S*D*_*D*_ is the difference of the paired samples values, and is classified as null (0.0), minimum (0.1−0.2), low (0.21−0.49), medium (0.5−0.79), and high (up to 0.8) (25). The Spearman correlations tests were used to test possible relationships between BMI, physical activity, strength, diet quality, serum markers, and microbiota composition both at T0 and T1. *R*-values were used to categorize the correlations as follows: false (*R* = | 0.09|), weak (*R* = | 0.10−0.39|), moderate (*R* = | 0.40−0.70|), strong (*R* = | 0.71−0.80|), very strong (*R* = | 0.80−0.99|), and perfect (*R* = | 1.00|) (26). Where necessary, the magnitude of the correlations was tested using the traditional Fisher-Z-approach and the algorithm of Olkin and Pratt (27), in accordance with Eid et al. (28). The significance level was established at 5% for all analyses (*p* < 0.050).

## Results

The mean age of participants was 66 years, and their mean years of schooling was 9.9. Most of them were single or widowed, and lived alone, as shown in Table 1.

**TABLE 1:** Sociodemographic characteristics of participants (*n* = 15).

		Participants
Age (mean ± standard deviation)		66.0 ± 6.2
Years of study (n/%)		
	1−4	2 (13.3)
	5−8	1 (6.6)
	9−11	3 (18.9)
	>12	9 (60.0)
Marital status		
	Single	7 (46.7)
	Court	2 (13.3)
	Married	2 (13.3)
	Widowed	3 (20.0)
	Divorced/Separated	1 (6.6)
Whom do you live?		
	Alone	7 (46.7)
	Spouse	2 (13.3)
	Son(s) and Grandchildren	4 (26.7)
	Brother/Sister(s)	2 (13.3)

### Body mass index and physical activity assessment

There was a significant increase of BMI in the 6 months following physical distancing (*p* = 0.030), although with a low effect size (Cohen’s *d* = 0.2), since BMI was 30.2 ± 4.0 kg/m^2^ at T0 and 30.9 ± 3.9 kg/m^2^ at T1. At T0, 26.8% of the participants had BMI within the range deemed appropriate by the World Health Organization, 26.6% of the participants were obese (BMI greater than or equal to 30), 46.6% were overweight (BMI greater than or equal to 25). After 6 months of physical distancing due to the COVID-19 pandemic, 50% of the participants were classified as obese and 50% as overweight (29).

**FIGURE 1:**
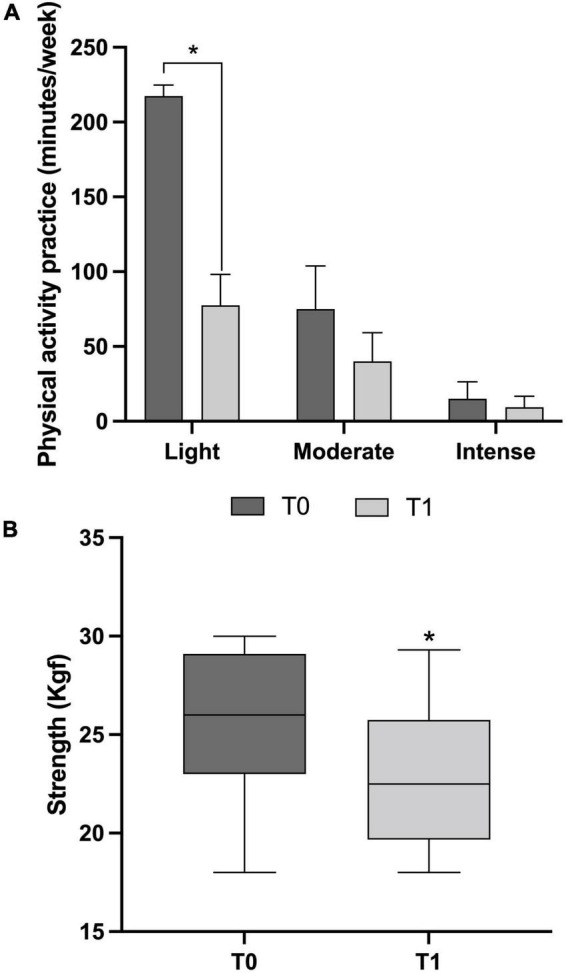
Physical activity practice (minutes/week) and Strength (kgf) before (T0) and after 6 months of physical distancing (T1) of Brazilian older women (*n* = 15). **(A)** Mean and standard deviation values for light (T0: 217.50 ± 47.13 and T1:77.50 ± 20.70), moderate (T0: 75.00 ± 28.91 and T1: 40.00 ± 19.18), and intense (T0: 15.00 ± 11.34 and T1: 9.37 ± 7.47), physical activity practice. **(B)** The boxplots show the median, minimum and maximum values for handgrip strength (T0: 27.05, min 22.50, max 30.00; T1: 22.50, min 18.00, max 29.03). * represents the difference between groups (paired Student *t*-test, *p* < 0.005).

As expected, the IPAQ analysis showed a significant decrease, with strong effect size, in light physical activity at T1 when compared to T0 (*p* < 0.001, Cohen’s *d* = 4.11), presenting a reduction of 140 min/week (Figure 1A). No differences were detected for moderate (*p* = 0.375, Cohen’s *d* = 0.54) and intense physical activity (*p* = 0.197, Cohen’s *d* = 0.22), though a medium and low effect size may be reported for moderate and intense physical activity, respectively. The participants showed a reduction of 35 min/week for moderate and 5.7 min/week for intense physical activity in T1 (Figure 1A). In addition, strength significantly decreased (*p* < 0.001, Cohen’s *d* = 1.06), from 26.5 ± 2.9 kgf at T0 to 23.1 ± 3.7 kgf in T1 (Figure 1B).

### Food intake and diet quality

Regarding the food intake and diet quality assessed, no statistically significant differences were observed for total DST and its partial scores, as well as for fruit and vegetable and processed meat (*p* > 0.050, Table 2). However, 57.0% of the older women reduced their consumption of fruits and vegetables, and 14.5% increased their consumption of processed meat (data not shown). A significant increase was detected for nutritional supplements, since 40.0% of participants reported the inclusion of vitamin D at T1 (*p* = 0.008, Cohen’s *d* = 1.22). It is important to mention that the effect size was high for non-protective foods consumption at T1 (Cohen’s *d* = 0.86), while lower or minimum effects were detected for other categories.

**TABLE 2:** Mean ± standard deviation of total Dietary Screening Tool and its parts scores of the participants (*n* = 15) before (T0) and 6 months of physical distancing (T1).

		Mean ± Standard deviation	*t*	*p-*Value	Cohen’s *d*
Dietary screening tool scores	T0	57.30 ± 9.26	0.92	0.373	0.36
	T1	60.79 ± 10.82			
Protective foods scores	T0	39.07 ± 9.28	0.06	0.948	0.03
	T1	38.86 ± 8.07			
Non-protective foods scores	T0	19.36 ± 2.56	1.50	0.156	0.86
	T1	22.00 ± 5.68			
Fruit and vegetables scores	T0	19.43 ± 6.81	0.14	0.893	0.06
	T1	19.07 ± 5.28			
Processed meat scores	T0	2.93 ± 1.82	0.23	0.821	0.11
	T1	3.14 ± 2.21			
Supplement scores	T0	0.00 ± 0.00	3.12	0.008*	1.22
	T1	2.14 ± 2.57			

The values of paired Student t-test, p-Value and Cohen’s d were also shown. * represents statistical significance (p < 0.050).

### Immunological (IL-2, IL-5, IL-6, IL-10, IFN-γ, and TNF-α), neurological (BDNF and cortisol), and aging-associated (VEGF, IGF-1, klotho, and TSLP) serum markers

**FIGURE 2:**
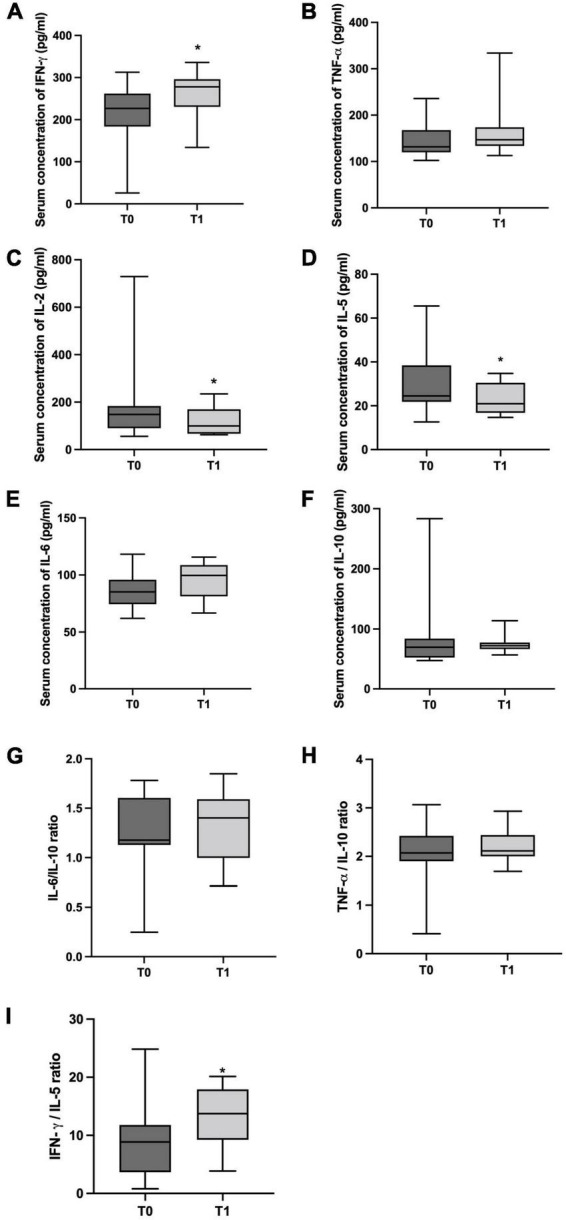
The boxplots show the median/minimum-maximum values for serum concentration of cytokines (pg/ml). **(A)** interferon- gamma (IFN-γ, T0: 219.20/25.80-288.40 and T1: 278.00/134.00-336.00), **(B)** tumor necrosis factor- alpha (TNF-α, T0: 136.00/102.20-236.00 and T1: 146.90/112.90-333.90), **(C)** Interleukin (IL)-2 (T0: 148.30/55.75-267.00 and T1: 99.75/62.50-234.50), **(D)** IL-5 (T0: 24.83/21.70-52.86 and T1: 20.93/14.70-34.77), **(E)** IL-6 (T0: 98.79/70.31-118.20 and T1: 99.58/66.63-115.70), **(F)** IL-10 (T0: 71.13/47.74-283.40 and T1: 72.3/56.93-113.90) and **(G)** IL-6/IL-10 (T0: 1.32/0.25-1.78 and T1: 1.40/0.71-1.85), **(H)** TNF-α/IL-10 (T0: 2.14/0.41-3.07 and T1: 2.11/1.69-2.93), **(I)** IFN-γ/IL-5 (T0: 8.63/0.81-12.33 and T1: 13.75/3.85-20.14) ratios before (T0) and after 6 months of physical distancing (T1). * differences between groups (Wilcoxon test, *p* < 0.050).

The serum concentration of IFN-γ was significantly higher (*p* = 0.009, Figure 2A), while IL-2 and IL-5 concentrations were lower at T1 in comparison to T0 (*p* = 0.050, and *p* = 0.032, Figures 2C,D). Furthermore, the effect size was high for IFN-γ (Cohen’s *d* = 0,94) and low for IL-2 (Cohen’s *d* = 0.21) and IL-5 (Cohen’s *d* = 0.21), respectively. No statistical differences and low effect size were detected for IL-6 (*p* = 0.637 and Cohen’s *d* = 0.38, Figure 2E), IL-10 (*p* = 0.534 and Cohen’s *d* = 0.27, Figure 2F), and TNF-α (*p* = 0.413 and Cohen’s *d* = 0.31, Figure 2B). To investigate if the inflammatory status and T helper response pattern had changed after 6 months of physical distancing, the ratio between IL-6/IL-10, TNF-α/IL-10 and IFN-γ/IL-5 were also performed. Although there were no differences between inflammatory ratios [IL-6/IL-10 (*p* = 0.831 and Cohen’s *d* = 0.27, Figure 2G) and TNF-α/IL-10 (*p* = 0.831 and Cohen’s *d* = 0.15, Figure 2H)], the IFN-γ/IL-5 ratio (Figure 2I) was increased in T1 in comparison to T0 (*p* = 0.007 and Cohen’s *d* = 1.34).

**FIGURE 3:**
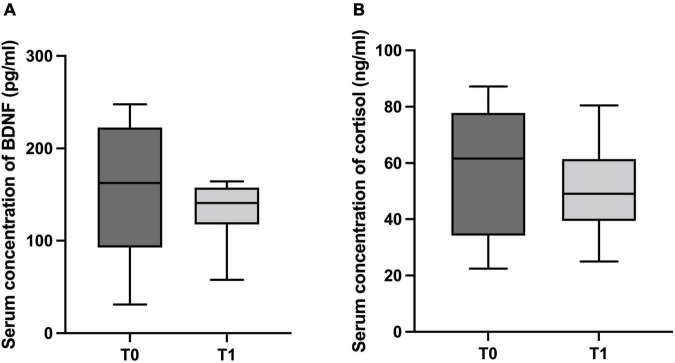
The boxplots show the median/minimum-maximum values for serum concentration of neurological markers before (T0) and after 6 months of physical distancing (T1). **(A)** Brain-derived neurotrophic factor (BDNF) in pg/ml (T0: 201.00/84.33-247.70 and T1: 141.00/57.67-164.30); **(B)** Cortisol in ng/ml (T0: 60.34/22.44-85.96 and T1: 51.87/24.98-80.45).

Although no significant differences between T0 and T1 regarding BDNF and cortisol serum concentrations have been detected (*p* = 0.123 and *p* = 0.845, respectively), a medium and high effect size was noted for BDNF and cortisol (Cohen’s *d* = 0.6 and 0.96, respectively), as illustrated in Figures 3A,B.

**FIGURE 4:**
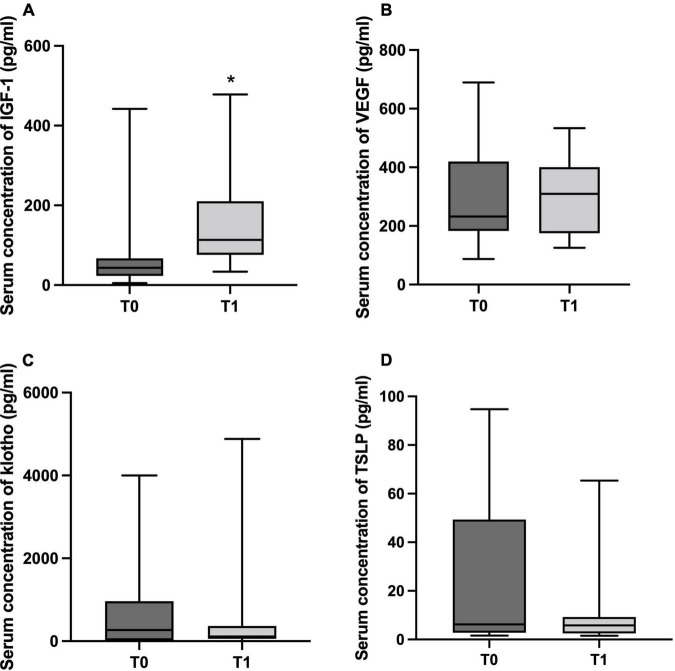
The boxplots show the median/minimum-maximum values for serum concentration (pg/ml) of aging-associated markers before (T0) and after 6 months of physical distancing (T1). **(A)** insulin-like growth factor-1 (IGF-1, T0: 36.63/4.88-441.90 and T1: 113.30/33.66-478.20), **(B)** vascular endothelial growth factor (VEGF, T0: 230.40/87.30-548.90 and T1: 309.40/125.30-533.90), **(C)** klotho (T0: 267.50/0-4002.00 and T1: 115.5/0-4882.00), and **(D)** thymic stromal lymphopoietin (TSLP, T0: 5.51/1.62-94.73 and T1: 5.61/1.57-65.35). * differences between groups (Wilcoxon test, *p* < 0.05).

Regarding aging-associated serum markers, there was a significant increase in IGF-1 concentration at T1 (*p* = 0.001; Figure 4A), considered as medium effect size (Cohen’s *d* = 0.71). No differences between T0 and T1 were detected for VEGF, klotho and TSLP concentrations (*p* = 0.174, *p* = 0.637, and *p* = 0.275, respectively). Nonetheless, a medium effect size was also reported for VEGF (Cohen’s *d* = 0.56, Figure 4B), while low effect size was observed for klotho and TSLP (Cohen’s *d* = 0.31 and 0.30, Figures 4C,D, respectively).

### Microbiota composition

The hypervariable region V3-V4 of bacterial *16SrRNA* gene was sequenced obtaining an average of 382,232 ± 91,274 readings with rarefaction observed at nearly 20,000 readings. Raw data are deposited in a BioProject in the NCBI Sequence Read Archive: PRJNA857193.

**FIGURE 5:**
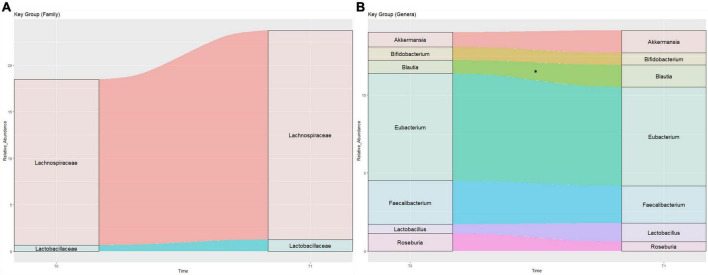
Sankey plot displays the proportions of relative abundance of bacterial groups in Family level **(A)** and Genera level **(B)** in two times, T0 and T1. *Differences between groups at T0 and T1 (Wilcoxon test, *p* < 0.05).

Comparisons between T0 and T1 were performed for Firmicutes and Bacteroidetes Phyla, Lactobacillaceae and Lachnospiraceae families and to *Roseburia* spp., *Faecalibacterium* spp., *Akkermansia* spp., *Eubacterium* spp., *Lactobacillus* spp., *Blautia* spp. and Bifidobacterium spp. (Figure 5). Although no changes were seen for most of the aforementioned microorganisms (*p* > 0.050), higher abundance and medium effect size was found for *Blautia* spp. at T1 (*p* = 0.035, Cohen’s *d* = 0.59). In addition, the Firmicutes/Bacteroidetes ratio (F/B) was calculated and no significant difference between T0 and T1 was detected (4.36 ± 5.35 at T0 and 2.29 ± 1.65 at T1, *p* = 0.668). It’s relevant to mention that medium effect size was also reported to Lachnospiraceae family, *Roseburia* spp., and F/B (Cohen’s *d* = 0.54), while Firmicutes (Cohen’s *d* = 0.21) and Bacteroidetes Phyla (Cohen’s *d* = 0.29), as well as *Akkermansia* spp. (Cohen’s *d* = 0.36) exhibited low effect size.

### Associations between physical parameters, diet quality, serum markers, and fecal microbiota composition

The Spearman correlations for BMI showed no significant correlations with diet quality and serum markers in T0 (Table 3), and moderate negative correlations were detected to Lachnospiraceae (*p* = 0.032, R^2^ = 0.31), *Roseburia* spp. (*p* = 0.016, R^2^ = 0.38), and *Faecalibacterium* spp. (*p* = 0.036, R^2^ = 0.30) for the same period (Table 4). Regarding T1, there was only one positive correlation between BMI and cortisol (*p* = 0.037, R^2^ = 0.50).

**TABLE 3:** Associations of Body Mass Index (BMI), physical activity, strength, fruit and vegetables, and serum markers by Spearman correlations analysis before (T0) and 6 months of physical distancing (T1).

Physical ×	T0				T1			
Diet ×	BMI	Physical activity		Strength	BMI	Physical activity		Strength
Serum markers		Light	Moderate			Light	Moderate	
Fruits and vegetables	−0.06 (0.838)	−0.10 (0.761)	−0.25 (0.456)	0.52 (0.050)*	−0.50 (0.154)	−0.24 (0.600)	−0.48 (0.288)	0.12 (0.708)
Cortisol	−0.18 (0.566)	0.09 (0.804)	0.04 (0.921)	0.35 (0.241)	0.71 (0.037)*	0.09 (0.933)	0.54 (0.267)	−0.25 (0.470)
IL-2	0.27 (0.338)	−0.37 (0.261)	−0.05 (0.883)	−0.58 (0.033)*	−0.07 (0.854)	−0.09 (0.933)	−0.54 (0.267)	−0.02 (0.950)
TNF-α /IL-10	0.24 (0.406)	−0.25 (0.452)	0.38 (0.244)	−0.60 (0.026)*	−0.28 (0.454)	0.00 (1.000)	−0.68 (0.200)	−0.22 (0.310)
VEGF	−0.21 (0.461)	−0.34 (0.306)	0.15 (0.663)	−0.68 (0.009)*	0.53 (0.150)	−0.44 (0.433)	0.34 (0.533)	0.49 (0.380)
IGF-1	−0.14 (0.634)	−0.41 (0.213)	0.79 (0.006)*	0.08 (0.776)	0.07 (0.868)	0.62 (0.244)	0.13 (0.800)	−0.51 (0.990)
Klotho	0.35 (0.224)	0.79 (0.006)*	0.10 (0.766)	−0.05 (0.863)	−0.11 (0.784)	0.79 (0.100)	0.30 (0.600)	−0.64 (0.590)
TSLP	−0.14 (0.629)	−0.60 (0.056)	0.34 (0.304)	−0.70 (0.007)*	0.09 (0.819)	0.09 (0.933)	0.54 (0.267)	−0.71 (0.510)

The values of R and p (in brackets) were presented, and * represents statistical significance (p < 0.050). IL, Interleukin; TFN-α, Tumor necrosis factor-alpha; VEGF, Vascular endothelial growth factor; IGF-1, insulin-like growth factor-1; TSLP, Thymic stromal lymphopoietin-TSLP.

**TABLE 4:** Associations of BMI, physical activity, strength, and microbiota composition by Spearman correlations analysis before (T0) and 6 months of physical distancing (T1).

Physical parameters ×	T0				T1			
Microbiota composition	BMI	Physical activity		Strength	BMI	Physical activity		Strength
		Light	Moderate			Light	Moderate	
*Akkermansia* spp.	−0.04 (0.895)	−0.23 (0.487)	−0.22 (0.512)	0.53 (0.045)*	0.43 (0.218)	0.13 (0.757)	−0.45 (0.264)	−0.52 (0.084)
*Blautia* spp.	0.19 (0.483)	−0.02 (0.954)	0.065 (0.848)	0.52 (0.050)*	0.46 (0.186)	0.18 (0.668)	0.24 (0.561)	−0.06 (0.847)
*Faecalibacterium* spp.	−0.55 (0.036)*	−0.63 (0.043)*	0.54 (0.090)	0.18 (0.516)	0.22 (0.542)	−0.01 (0.988)	0.72 (0.056)	−0.36 (0.247)
*Roseburia spp.*	−0.62 (0.016)*	−0.25 (0.452)	−0.22 (0.512)	0.26 (0.351)	0.56 (0.093)	0.27 (0.519)	0.34 (0.404)	−0.14 (0.662)
Bacteroidetes phylum	0.32 (0.246)	0.12 (0.713)	−0.065 (0.851)	−0.59 (0.023)*	−0.13 (0.723)	0.34 (0.405)	−0.16 (0.695)	−0.09 (0.788)
Firmicutes phylum	−0.03 (0.920)	−0.04 (0.918)	−0.296 (0.371)	0.59 (0.024)*	0.46 (0.181)	−0.35 (0.389)	0.34 (0.404)	−0.42 (0.167)
Firmicutes/Bacteroidetes	−0.06 (0.036)*	−0.19 (0.578)	−0.053 (0.882)	0.12 (0.673)	−0.34 (0.330)	0.50 (0.214)	−0.76 (0.040)*	−0.03 (0.919)
Lachnospiraceae family	−0.56 (0.032)*	−0.39 (0.230)	−0.062 (0.530)	0.46 (0.790)	0.07 (0.845)	−0.09 (0.776)	−0.15 (0.72)	0.06 (0.934)

The values of R and p (in brackets) were presented, and * represents statistical significance (p < 0.050).

Regarding the different physical activity levels, no correlations were detected with food intake and diet quality for both periods (*p* > 0.050, Supplementary Table 1). Nevertheless, light physical activity presented strong positive correlation with klotho (*p* = 0.006, R^2^ = 0.62), while moderate physical activity correlates negatively with IL-10 (*p* = 0.038, R^2^ = 0.41) and positively with IGF-1 (*p* = 0.006, R^2^ = 0.63) at T0 (Table 3). Correlations between physical activity and microbiota were detected only for light levels of physical activity, which negatively correlated with *Faecalibacterium* spp. at T0 (*p* = 0.043, R^2^ = 0.39). A strong negative correlation was observed at T1 between moderate physical activity and Firmicutes/Bacteroidetes ratio at T1 (*p* = 0.042, R^2^ = 0.58, Table 4).

Only one positive correlation was found between strength and fruit and vegetables intake at T0 (*p* = 0.050, R^2^ = 0.27). However, there were negative correlations between strength and serum markers at T0, as follows: IL-2 (*p* = 0.033, R^2^ = 0.33), VEGF (*p* = 0.009, R^2^ = 0.46), TSLP (*p* = 0.007, R^2^ = 0.49), and TNF-α/IL-10 ratio (*p* = 0.026, R^2^ = 0.36). Strength also positively correlates with *Akkermansia* spp. (*p* = 0.045, R^2^ = 0.28), *Blautia* spp. (*p* = 0.050, R^2^ = 0.27), and Firmicutes phylum (*p* = 0.024, R^2^ = 0.34); and negatively with Bacteroidetes phylum (*p* = 0.023, R^2^ = 0.35).

Some significant correlations were also detected between dietary scores and neurological markers. At T0, DST total score correlated positively and moderately with BDNF (*p* = 0.043, R^2^ = 0.30); however, at T1, strong and negative correlations can be observed in relation to DST total score (*p* = 0.016, R^2^ = 0.56) and fruit and vegetables (*p* = 0.042, R^2^ = 0.435), as shown in Table 5. The r→z Fisher transformation demonstrated that these correlations were stronger in T1 (*z* = 2.89, *p* = 0.002). Interestingly, cortisol concentration presented a strong negative correlation with fruit and vegetables intake (*p* = 0.022, R^2^ = 0.52) at T1.

**TABLE 5:** Association of Dietary Screening Tool (DST), its parts scores and serum markers by Spearman correlations analysis before (T0) and 6 months of physical distancing (T1).

		T0			T1	
		
	DST	Fruit and vegetables	Processed meat	DST	Fruit and vegetables	Processed Meat
BDNF	0.55 (0.043)*	0.38 (0.183)	0.20 (0.485)	0.75 (0.016)*	−0.66 (0.042)*	0.14 (0.716)
Cortisol	0.50 (0.087)	0.49 (0.081)	0.35 (0.485)	0.06 (1.000)	−0.72 (0.022)*	0.27 0.464
IL-2	−0.04 (0.901)	−0.36 (0.203)	0.18 (0.534)	0.61 (0.067)	−0.18 (0.614)	0.54 (0.119)
IL-5	0.17 (0.547)	−0.10 (0.739)	−0.53 (0.854)	0.50 (0.144)	−0.33 (0.353)	0.51 (0.149)
IL-6	−0.07 (0.843)	0.17 (0.636)	0.27 (0.480)	−0.43 (0.212)	−0.32 (0.361)	−0,55 (0.109)
IL-10	−0.07 (0.857)	0.24 (0.494)	−0.67 (0.046)*	−0.04 (0.918)	−0.22 (0.542)	−0.08 (0.872)
IFN-γ	−0.73 (0.020)*	0.000 (1.000)	−0.27 (0.480)	−0.37 (0.296)	−0.11 (0.766)	−0.35 (0.337)
TFN-α	−0.04 (0.924)	0.34 (0.331)	−0.18 (0.654)	−0.13 (0.733)	−0.42 (0.227)	−0.19 (0.612)
VEGF	0.39 (0.264)	−0.65 (0.046)*	0.81 (0.009)*	−0.09 (0.811)	−0.16 (0.649)	0.23 (0.542)
IL-6/IL-10	−0.27 (0.440)	−0.44 (0.198)	0.81 (0.009)*	−0.19 (0.607)	−0.97 (0.792)	−0.54 (0.119)
TNF-α/IL-10	−0.20 (0.575)	−0.32 (0.369)	0.72 (0.029)*	−0.13 (0.733)	0.30 (0.391)	−0.27 (0.464)
IFN-γ/IL-5	−0.51 (0.139)	0.13 (0.725)	−0.40 (0.268)	−0.62 (0.060)	0.18 (0.624)	−0.74 (0.025)*

The values of R and p (in brackets) were presented, and * represents statistical significance (p < 0.050). BDNF, Brain derived-neurotrophic factor; IL, Interleukin; IFN-γ, Interferon gamma; TFN-α, Tumor necrosis factor-alpha; VEGF, Vascular endothelial growth factor.

Correlations between immunological markers and diet quality (DST total score, fruits and vegetables, and processed meat) were clearly observed at T0 (Table 5). Positive correlations between processed meat consumption and serum markers (IL-6/IL-10, TNF-α/IL-10, and VEGF) were detected; negative correlations between fruits and vegetables intake and the VEGF marker were also detected. However, 6 months following the pandemic period (T1), a negative correlation was observed between intake of processed meat and the IFN-γ/IL-5 ratio. Unexpectedly, no correlations were found between dietary marker scores and microbiota abundances in both periods.

**FIGURE 6:**
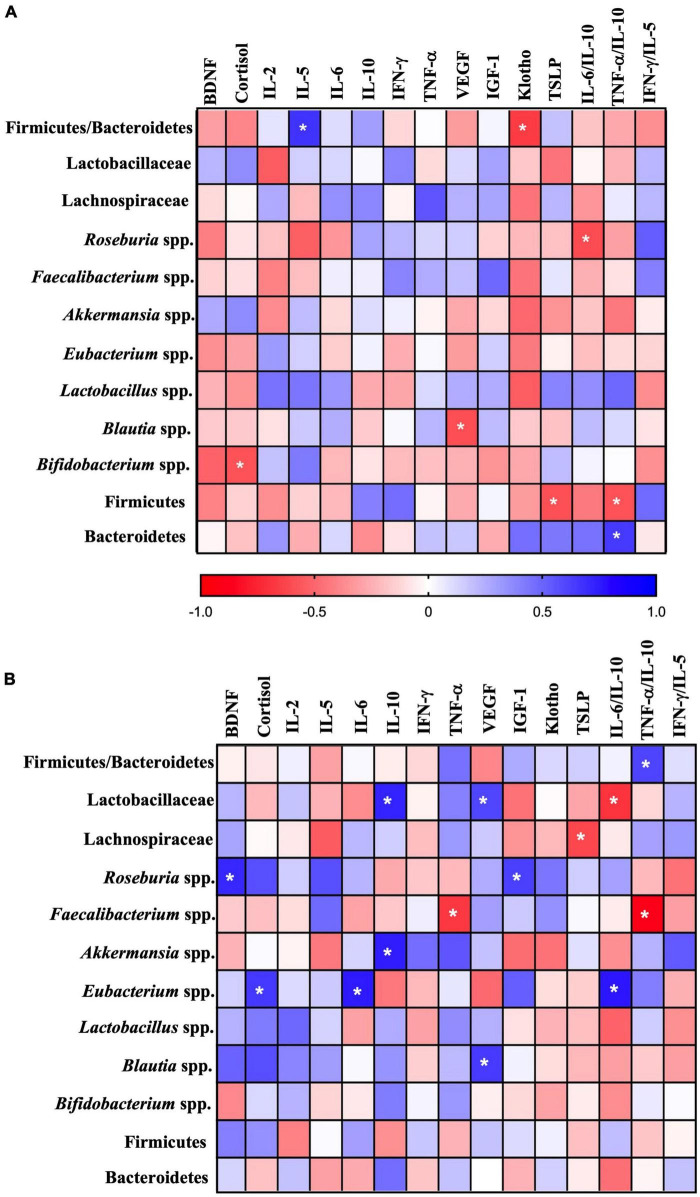
Spearman correlation between serum markers and microbiota composition before (T0, **A**) and after 6 months of physical distancing (T1, **B**). The heat maps illustrate the *R*-value, in accordance with the intensity of colors (horizontal bar). Red represents negative correlations while blue represents positive correlations. * illustrates the significant correlations (*p* < 0.05). BDNF, brain-derived neurotrophic factor; IL, Interleukin; IFN-γ, interferon-gamma; TNF-α, tumor necrosis factor-alpha; VEGF, vascular endothelial growth factor; IGF-1, insulin-like growth factor-1; TSLP, thymic stromal lymphopoietin.

All correlations made between serum markers (IL-2, IL-5, IL-6, IL-10, IFN-γ, TNF-α, BDNF, cortisol, VEGF, IGF-1, klotho, and TSLP) and microbiota composition are shown in Figure 6, and significant associations are detailed hereafter. At T0, positive and moderate correlations were detected between F/B ratio and IL-5 (*p* = 0.015, R^2^ = 0.45), and TNF-α/IL-10 ratio and the Bacteroidetes phylum (*p* = 0.019, R^2^ = 0.42). In addition, several negative correlations were found: cortisol and *Bifidobacterium* spp. (*p* = 0.040, R^2^ = 0.34), VEGF and *Blautia* spp. (*p* = 0.034, R^2^ = 0.36), klotho and F/B (*p* = 0.016, R^2^ = 0.43), TSLP and Firmicutes (*p* = 0.036, R^2^ = 0.35), IL-6/IL-10 ratio and *Roseburia* spp. (*p* = 0.032, R^2^ = 0.36), TNF-α/IL-10 ratio and Firmicutes (*p* = 0.041, R^2^ = 0.34).

Regarding T1, positive correlations were found between: BDNF and *Roseburia* spp. (*p* = 0.017, R^2^ = 0.50), cortisol and *Eubacterium* spp. (*p* = 0.034, R^2^ = 0.56), IL-6 and *Eubacterium* spp. (*p* = 0.010, R^2^ = 0.56), IL-10 with the Lactobacillaceae family (*p* = 0.015, R^2^ = 0.43), and *Akkermansia* spp. (*p* = 0.011, R^2^ = 0.56), VEGF with the Lactobacillaceae family (*p* = 0.050, R^2^ = 0.37) and *Blautia* spp. (*p* = 0.01, R^2^ = 0.42), IGF-1 and *Roseburia* spp. (*p* = 0.037, R^2^ = 0.40), IL-6/IL-10 ratio and *Eubacterium* spp. (*p* = 0.009, R^2^ = 0.58), TNF-α/IL-10 and F/B ratios (*p* = 0.049, R^2^ = 0.38). In addition, negative correlations were found between: TNF-α and *Faecalibacterium* spp. (*p* = 0.025, R^2^ = 0.46), TSLP and the Lachnospiraceae family (*p* = 0.044, R^2^ = 0.40), TNF-α/IL-10 and *Faecalibacterium* spp. (*p* = 0.001, R^2^ = 0.58), while the proportion IL-6/IL-10 correlates with *Eubacterium* spp. (*p* = 0.001, R^2^ = 0.79) and Lactobacillaceae family (*p* = 0.009, R^2^ = 0.49).

## Discussion

Social interactions are proposed to be a basic human need, just as food consumption or sleep. Living generations had never experienced situations of lockdown and physical/social distancing like those imposed by the COVID-19 pandemic. Efforts to contain the spread of COVID-19 have required sudden, often mandatory physical distancing, removing many regular sources of social connection from people’s lives. Such measures are likely to have a substantial effect, not only on the economy and society, but also on individual mental health and wellbeing due to factors such as reduced contact with other people (30). Indeed, feeling insufficiently connected to others is associated with profound, lasting negative consequences on people’s physical and mental health, and may even lead to increased mortality. The effects of such social contact deprivation will possibly extend beyond the physical distancing period and might affect the population for years to come. Besides the psychological implications, physical parameters must be considered to better understand its health impact. Modifications of lifestyle of the world population seem to promote negative health-related psychosocial outcomes, mainly associated with physical activity level, diet and sleep quality (31, 32). It is important to consider that these impacts are directly linked to specific characteristics of the studied population, such as country/region location, age, health and social status, income, political and economic stability, among many other aspects.

This study evaluated the effects of 6 months of physical distancing on Brazilian older women regarding eating habits, neurological markers (BDNF, cortisol), cytokines (IL-2, IL-5, IL-6, IL-10, IFN-γ, and TNF-α), aging-associated markers (VEGF, IGF-1, klotho, and TSLP) and specific groups of fecal microbiota. To the best of our knowledge, the present study is the first one including assessment of immunological and microbiome composition effects, as well physical activity, and food intake after physical distancing due to COVID-19 for older people living in a large urban center.

Overall, health benefits promoted by regular physical activity practice have been fully proven by several studies (33–38). Besides preventing physical and cognitive decline (33) and improving well-being (34), physical activity enhances glycemic control (33), immunological response (35), and cardiovascular system function (36, 37), contributing to decreased mortality (37, 38). In addition, for older adults, physical activity practice prevents falls and frailty by improvement of muscular strength and balance (39, 40). The older women included in this study presented an increase in BMI, and a decrease in physical activity practice and strength after 6 months of physical distancing, which could promote an increase of morbimortality, as suggested by Peçanha et al. (41). Wang et al. (42) studying 2,289 subjects older than 18 years in China after 77 days of physical distancing, reported lower physical activity, and higher length of sitting and lying down time. Furthermore, Marcos-Pardo et al. (43) demonstrated increased levels of LDL, Non-HDL lipids and reduced levels of HDL, muscle strength, and femoral bone mass densitometry after 13 weeks of physical distancing due to the COVID-19 pandemic in Spanish older women (60−70 years, *n* = 30). Although BMI was not significantly modified, total and trunk fat mass increased, which could contribute to increases in cardiovascular risk in those older women. Therefore, similarly to the findings of the present study, physical distancing seems to negatively affect the health of the older population, since such BMI increase and strength decrease could be associated with fat tissue accumulation.

Considering that food choice responds to physiological, emotional, and social stimuli, the 6 months of physical distancing may have impacted food intake in several aspects. Regarding diet quality, some studies observed no modifications on eating habits during physical distancing (44), while others reported beneficial and healthy changes, or described harmful changes in diet quality (8, 10, 45, 46). Sanchez-Sanchez et al. (44) showed that 46.1% of 4,500 Italians reported not having changed their eating habits, but 37.2% reported a worsened diet quality. Ruiz-Roso et al. (45) observed that older people increased their consumption of sweet drinks and snacks, while Renzo et al. (10) found an increase in the practice of cooking at home and hypothesized that this habit may have led to the use of natural and healthier foods. In the present study, an increased consumption of non-protective foods (high effect-size) was reported after 6 months of physical distancing, while minimal changes were detected for other categories, such as fruits and vegetables. Discordant results were described by Wang et al. (42) who found an improvement in the diet quality associated with higher intake of fruits and vegetables. Some possible explanations for these differences are the follow-up time and studied population, since the present study considered 6 months of physical distancing in older women, while the aforementioned studies were performed using different sample periods, in different countries, and included subjects from different age groups, starting at 12-year-olds. Moreover, countless external factors could be mentioned, such as income, public health system, social support, regional restriction rules, access to grocery stores or food delivery systems, number of cohabitants, restricted-food habits, among many others.

It is important to mention that alterations in eating habits vary greatly in relation to the social and economic characteristics of the studied population. In developing countries, an increase in food insecurity was observed during the COVID-19 pandemic, while in developed countries the risk was associated with an increase in obesity (47). Although Brazil is considered a developing country, the participants of the present study live in a high-medium class traditional district that has an elevated Human Development Index (HDI)^1^. In addition, the participants presented a high schooling level, that may impact our findings and does not represent most of the Brazilian population, who may be more harmfully impacted by physical distancing. Smaira et al. (48) showed, during 4 months of physical distancing due to the COVID-19 pandemic, that Brazilian women (>18 years) with normal BMI and high education level, have some eating habits and food choices associated with an unhealthy diet, including high energy and carbohydrate consumption, increased ultra-processed food consumption, and reduced unprocessed/minimally processed foods consumption.

Concentrations of some cytokines were altered after 6 months of physical distancing. Data showed an increase of IFN-γ and the IFN-γ/IL-5 ratio and decrease in IL-2 and IL-5. IFN-γ is associated with adaptive immune response development through enhancement of MHC I and II expression on Antigen Presenting Cells and phagocytic functions of macrophages (49). Moreover, chronic activation of mice microglia with IFN-γ has been associated with impaired adult hippocampal neurogenesis and leads to depression-like behaviors and cognitive deficits (50). Fard et al. (51) investigated the relationship between several domains of cognitive function and many cytokines in healthy older adults (60−75 years, *n* = 286), and described a strong association between higher concentrations of IFN-γ and low continuity of attention scores, while the relationship between IFN-γ and speed of memory was influenced by the BMI. Authors suggested that cognitive function can be affected differently by higher immune activity, characterizing a protective response for subjects with normal BMI, or damaging to overweight or obese subjects. IL-2 is an essential cytokine to maintain Treg cells controlling immune responses and to stimulate T cells promoting immune responses (52). Decreased concentrations of IL-2 in older adults have been associated with immunosenescence (51, 52) and seemed to promote Th17 differentiation in aged mice (53). Kleiner at al. (54) reported undetectable levels of IL-5 in serum of healthy subjects, however, this cytokine was associated with the pathophysiology of asthma. On the other hand, IL-5 presents functions associated with reduced aging-associated neuroinflammatory processes and improvement of cognitive decline observed in older adults (55). Taken together, our findings may suggest that the alterations in IFN-γ, IL-2 and IL-5 after 6 months of physical distancing could affect neurological functions and inflammatory patterns in older women.

This hypothesis may be reinforced by the reduced concentration of BDNF found in participants after 6 months of physical distancing. BDNF is an important regulator of synaptic transmission and long-term potentiation in the hippocampus and in other brain regions, suggesting an involvement in adult neurogenesis. In addition, BDNF is a crucial molecule for the central nervous system’s ability to regenerate and adapt to possible damage (56). There is some evidence about BDNF expression in skeletal muscle, cardiac, liver, and adipose cells, suggesting an association between beneficial physical activity practice and healthy diet on neuroplasticity mediated by BDNF (57). Furthermore, the increase of BDNF expression may be promoted by external stimuli, such as physical activity and caloric restriction (58, 59). However, further research is required to better understand the association between BMI, inflammation, and cognitive functions in older adults.

Several molecules have been studied as hallmarks of aging in biological systems, whom the IGF-1 signaling may be considered an evolutionarily conserved via that controls longevity and plays a major role in growth, differentiation, and metabolism, in response to environmental conditions and nutrient availability (59). A decrease in the IGF-1 via signaling has been associated with oxidative stress reduction and long-lived phenotypes in mice (60). Although the role of IGF-1 in human aging remains partially to be clarified, it has been speculated that lower levels of glucose, insulin, preserved insulin sensitivity and lower level of IGF-1 may represent key features to human longevity (61). In the present study, participants exhibited an increase in IGF-1 following 6 months of physical distancing, suggesting once again that lifestyle changes and environment stressors caused by the COVID-19 pandemic may have accelerated the aging-associated deleterious process.

Moreover, aging has been characterized by increased concentration of inflammatory markers, a clinical observation that has been termed “inflammaging” (62, 63). Dietary patterns rich in fruits and vegetables, a recognized source of vitamins and bioactive compounds, reduced serum levels of C-reactive protein and IL-6, important proinflammatory markers, as well as that of VEGF (64–67). Although in the present study no modifications have been observed in IL-6 and TNF-α, considered aging and disease-associated proinflammatory cytokines (68–71), the participants increased vitamin D supplementation, which could have helped to control their inflammatory status. Besides, Messina et al. (72) suggests the use of IL-6 and TNF-α inhibitor drugs as a possible therapy for COVID-19, justifying the importance of nutritional intervention to reduce the release of pro-inflammatory cytokines.

In the present study, associations between physical parameters, eating habits, serum markers, and microbiota were also performed. In relation to physical parameters and eating habits, only one positive correlation was seen between strength and fruit and vegetables consumption before physical distancing, which reinforces the importance of diet quality for older women. This finding, in accordance with other studies, showed a beneficial effect of fruit and vegetables rich diets on muscle strength in older women (73, 74). It was also observed that strength was negatively associated with IL-2, TNF-α/IL-10, VEGF, and TSLP before physical distancing, suggesting the importance of reducing inflammation and vascular damage to avoid strength loss in older women (75). Although IL-2 is not considered a classical inflammatory cytokine, its depletion may promote the absence of transcription factor STAT5A and consequent enhanced differentiation of Th17 cells, a subpopulation of T cells involved in the pathophysiology of inflammatory diseases (76). Also, IL-10 plays a role as an anti-inflammatory cytokine, besides associating with longevity, vascular protection, and improvement of endothelial dysfunction (77–79). VEGF may be considered essential to establish the vascular supply of different tissues, improving age-related conditions such as weight gain, hepatic steatosis, osteoporosis, inflammaging, and tumor (80). On the other hand, VEGF has also been implicated in the etiology of various neurological diseases, since it is also associated with vascular permeability, which can compromise nervous system and blood-brain barrier homeostasis, promoting the production of reactive oxygen species, migration of immunological cells and inflammation in humans and animals (81, 82), which contributes to vascular aging and age-related frailties and diseases. In turn, TSLP is a cytokine with diverse actions in immune homeostasis, regulating inflammatory response at barrier surfaces through increased proliferation of T cells and release of Th2 cyto- and chemokines from mast cells, innate lymphoid cells (ILCs), and macrophages. Thus, TSLP may be involved as initiator and propagator of allergic disease, such as atopic dermatitis, food allergy, and asthma, as well as cancer (83). Taken together, the aforementioned associations support the importance of maintaining strength in order to control inflammatory status in older women, reinforcing that adopting a healthy diet and physical activity practice is important.

In the current study, light physical activity presented strong positive correlation with klotho, while moderate physical practice correlated negatively with IL-10 and positively with IGF-1, a frailty marker. Klotho is an aging-associated marker responsible for regulating the activity of ion channels, and growth factor receptors, including insulin/IGF-1 receptors with anti-oxidative and anti-aging properties (84, 85). It has been reported that overexpression of klotho was associated with longevity (84), while lower level of expression caused by mutations in the human KLOTHO gene was associated with unhealthy aging (86, 87). Taken together, these findings suggest the beneficial effects of light physical activities, while moderate physical activity should be better investigated for older women included in this study, since it correlates with frailty markers.

Interestingly, all associations observed between physical activity and serum markers before physical distancing were not detected 6 months following, while a positive correlation was detected to BMI and cortisol. Since cortisol is an end-product of the hypothalamic-pituitary-adrenal axis associated with stress conditions in older adults (88), and it increases the search for pleasurable activities, such as the consumption of comfort foods rich in sugar and fat (89, 90); this finding could be associated with the stress period lived by participants of the present study.

Regarding eating habits and serum markers, there were negative correlations between the consumption of fruits and vegetables and VEGF, and between processed meat intake and IL-10; besides, there were strong positive correlations between the consumption of processed meat and inflammation and aging markers (IL-6/IL-10, TNF-α/IL-10, and VEGF) before the physical distancing. In part, the present results resemble other studies, in that they highlight the importance of *in natura* foods consumption and diminished intake of ultra-processed foods to reduce inflammatory and aging-associated markers (63–66, 91, 92). A 6 months following the physical distancing, the aforementioned correlations were also lost, and a negative correlation between the consumption of processed meat and IFN-γ/IL-5 ratio arose. Considering that inflammatory response is a dynamic network, continuously remodeling in consequence of the interaction between genes and environmental stimuli, it is feasible to speculate that compensatory mechanisms could have been activated in order to restore homeostasis (71, 93).

Once again, the correlations were modified in the evaluated periods since, before physical distancing, the DST score exhibited a positive correlation with BDNF, which was inverted in the 6 months following, when a strong negative correlation was detected. It is important to mention that the DST score may be used to assess overall diet quality and the total score is associated with a favorable dietary pattern (94). Thus, the negative correlation observed after 6 months could be explained by increased consumption of unhealthy foods by participants. In addition, a negative association between fruits and vegetables consumption and cortisol arose 6 months after physical distancing. Therefore, it could be speculated that stress conditions experienced by older women during the pandemic period promoted an increase in cortisol (high effect size), which could induce a higher intake of comfort foods to the detriment of healthy foods.

In general, gut microbiome has been related to health, and differences in its composition are observed in many disease conditions. It is important to consider that physical distancing because of the COVID-19 pandemic intersects with the hygiene hypothesis associated with decades-long decline in microbial diversity and ancestral microbes due to urban living, hygiene habits and use of antibiotics (17). In fact, these alterations contribute to microbiome imbalance with deleterious consequences for the host (16), increasing dysbiosis of gut microbiome in older adults. There is a paucity of information about the microbiome of South American populations, since most studies were performed in European, Asian and North American populations, that differ in genetic background, eating habits and environmental factors (19).

Despite significant inter-individual differences, the microbiome of healthy adults is dominated by Bacteroidetes-related Operational Taxonomic Units (OTUs) along with Firmicutes phylum (95, 96) with a striking variability between individuals, represented by an enormous range of the Firmicutes/Bacteroidetes (F/B) ratio (97). Conflicting data are presented about abundance of Bacteroidetes and Firmicutes phyla in relation to eating habits. Bacteroidetes phylum was already associated with lower consumption of animal products (98, 99), while no difference based on food choices was detected by other authors (96). Also, some studies showed that people with obesity have an elevated proportion of Firmicutes (100, 101), while other authors state that the most noticeable feature in the microbiota of the elderly is an increase in the relative proportion of Bacteroidetes (102); besides, others show decreased levels (103). Thus, it is difficult to associate F/B with a determined health status, obesity, or aging, due to broad differences in methods, interpretative bias, poor participants’ characterization regarding lifestyle-associated factors known to affect microbiota composition (104).

Microbiome modifications may be explained by altered environment due to physical distancing by the COVID-19 pandemic, since commensal bacteria exhibit flexibility to adapt to the host demand (105). In the current study, fecal microbiota dominant phyla were Firmicutes and Bacteroidetes and no statistical differences between their abundances and F/B ratio were detected before and after 6 months of physical distancing. However, the F/B ratio presents high variability among studies, since Mariat et al. (100) reported 0.6, while Vaiserman et al. (103) detected 1.42 for older adults and, in the present study, the F/B ratio decreased 52.5% (4.36 ± 5.35 to 2.29 ± 1.65) after physical distancing (medium effect size), which could be related to increase in age-associated Bacteroidetes, as described by Martinez et al. (102). This result is discordant with Aguilera et al. (19), which showed that F/B ratio in the pre-pandemic period was 1.01 ± 0.38 and, after five years (during the physical distancing period), it increased to 1.47 ± 0.96. The age of participants, methodological approaches and interval between sampled periods may justify the differences between these results.

It is widely reported that age affects gut microbiota composition (104), which is related to brain innate immunity (106), nervous system (107) and metabolic diseases (108). In the present study, in the period before the physical distancing, strength was negatively associated with Bacteroidetes abundance and positively associated with Firmicutes; also, Bacteroidetes abundance was positively correlated with TNF-α/IL-10 and F/B ratio with IL-5, corroborating that the increase in Bacteroidetes (102, 109) is associated with frailty and inflamed status of older adults (53). On the other hand, some negative correlations were observed, such as F/B ratio with klotho, Firmicutes with TSLP and TNF-α/IL-10, suggesting an association between higher F/B ratio, contributing to longevity and inflammation control in participants of the present study, since TSLP inhibits the Th1 and Th17 differentiation in the gut (110, 111). Further studies are necessary to understand the current unexpected results (102, 103).

Once more, after six months of distancing the correlations observed above were lost and an inversion of correlation between F/B ratio and TNF-α/IL-10 emerged, also, a negative correlation arose between F/B ratio and moderate physical activity. It is important to consider that the type of bacteria found in the gut of older people are quite variable, more so than in younger people (108), which makes it difficult to understand the real biological impact of these relationships.

Considering the abundance of Lactobacillaceae family, no correlations were detected in the initial period sampled, while positive correlations with IL-10 and VEGF, and negative association with IL-6/IL-10 were observed after 6 months of physical distancing, although *Lactobacillus* spp. showed no correlations with the studied parameters. It is important to point out that the Lactobacillaceae family includes many genera such as *Lactobacillus*, *Paralactobacillus*, and *Pediococcus.* Zheng et al. (112) showed extensive diversity of the genus *Lactobacillus*, an integral part of the microbiome with anti-inflammatory action (113). Some studies associated decrease in *Lactobacillus* spp. with frailty in older people and its role as probiotic enhancing anti-inflammatory functions (114, 115). In fact, these correlations between serum markers and the Lactobacillaceae family observed after 6 months of physical distancing could evidence the microbiota role on damage-control mechanisms.

Moreover, for the Lachnospiraceae family, negative correlations were detected with BMI and with TSLP before and after 6 months of distancing, respectively. It is well characterized that microbiome composition directly affects the balance of pro-inflammatory and anti-inflammatory responses in the gut. Bolte et al. (116) positively related the consumption of fast food and savory snacks with abundance of Lachnospiraceae bacteria in adults. This family is among the main producers of short-chain fatty acids and belong to the core of gut microbiota, colonizing the intestinal lumen from birth and increasing during life (117), but it has been shown that this bacterial family decreases along aging, which is associated with dysbiosis (109, 118, 119).

Regarding the studied genera, only *Blautia* spp. showed increased abundance after 6 months of physical distancing. This bacterium has been associated with Western diet, rich in fat, sugar, fast food, French fries, mayonnaise, and soft drinks (116). On the other hand, Ozato et al. (120) showed that *Blautia* is inversely associated with visceral fat area in women, and Benítez-Páez et al. (121) detected decreased incidence of *Blautia* species in obese children, which was more pronounced in cases of both obesity and insulin resistance and increased proinflammatory cytokines and chemokines, as IFN-γ and TNF-α in their feces. In the present study, *Blautia* positively correlated with strength and negatively correlated with VEGF before the distancing period, besides, it positively correlated with VEGF 6 months after the physical distancing. In a recent review, Vacca et al. (117) showed that *Blautia* and *Roseburia* are the genera most involved in the control of gut inflammatory processes, atherosclerosis, and maturation of the immune system by butyrate (SCFA). Once again, our findings showed an inverted correlation between the periods sampled and the putative intrinsic relationship between microbiota in an attempt to compensate an imbalance promoted by physical distancing.

No differences were found in *Roseburia* spp. abundance for the studied periods, however, after 6 months of distancing, the genus decreased (medium effect size), while the consumption of non-protective foods increased (high effect size). As stated by Bolte et al. (116), the consumption of nuts, oily fish, fruits, vegetables, cereals, and red wine is related to *Roseburia* abundance. Other studies (95, 117) demonstrated that fibers encourage the growth of this lactic acid and SCFA-producer bacteria with critical functions in the gut permeability. In the present study, it correlated negatively with BMI and IL-6/IL-10 before the physical distancing and correlated positively with BDNF and IGF-1 after 6 months of distancing. In addition, *Faecalibacterium* spp. correlated negatively with BMI and light physical activity before physical distancing and correlated negatively with TNF-α and TNF-α/IL-10 after 6 months.

Although the influence of physical activities on microbiota composition remains unclear in the literature, an increase of Bifidobacteriaceae has been previously shown in animal models after 5 weeks of physical training (122). In the present study *Bifidobacterium* showed a negative correlation with cortisol before physical distancing, suggesting its association with a lower stressor status supposedly present in this period. Thus, the loss of this correlation after 6 months of physical distancing could corroborate with the literature findings, which associates *Bifidobacterium* with anti-pathogenic and anti-inflammatory effects, besides cardiovascular protection. In addition, its lower abundance was associated with animal protein intake and inflammatory bowel diseases (95, 123).

To the best of our knowledge, there are no studies about changes in microbiota composition associated with physical distancing in older women, which hampers a better discussion of the present results. The closest study was performed by Aguilera et al. (19), that compared the gut microbiome of adults from Buenos Aires (Argentina) between 3 and 5 months of physical distancing. Although the initial samples were collected in 2016, the authors showed that five genera (*Desulfovibrio*, *Streptococcus*, *Anaerostipes*, *Oscillospiraceae*, and *Eubacterium*) were lost of the core microbiome, while three other genera (*Erysipelotrichaceae*, CAG-352 and *Akkermansia*) became part of the core microbiome after physical distancing. In the present study, no difference was observed to *Eubacterium* abundance before and after physical distancing, however, positive correlations with cortisol, IL-6 and IL-6/IL-10 were detected after 6 months of physical distancing. These results disagree with other studies, which have demonstrated that this genus, able to produce SCFA, is associated with adult consumption of protective foods (116) and is depleted in patients with bowel inflammatory diseases (124, 125). This apparent inconsistency could be associated with participants’ age and environmental stress conditions caused by the pandemic.

Bressa et al. (126) reported an increased abundance of *Akkermansia* spp., *Faecalibacterium* spp., and *Roseburia* spp., besides a lower abundance in Bacteroidetes phylum in active women compared to sedentary ones, which suggested a benefit of physical activity in microbiota composition. In accordance, in the present study, *Akkermansia* spp. correlates positively with strength and IL-10, before and after physical distancing, respectively. These results may be supported by other studies that showed that this genus contains the *Akkermansia muciniphila*, which has been linked to a healthy metabolic profile (68, 77) and exercise practice (68), and it has constantly been recognized as a promising candidate for the next generation of probiotics (78). However, this mucolytic bacterium has also been associated with production of endotoxins and induction of Th17 cells (116), misfolding of α-synuclein in enteroendocrine cells (127) and could be related to Parkinson disease (79). These findings exhibit the dissimilarities among studies and the difficulties to understand the biologic effects of microbiota composition in different host conditions.

Further studies are essential to understand the associations between physical activity, diet quality, immunological (IL-2, IL-5, IL-6, IL-10, IFN-γ, and TNF-α), neurological (BDNF and cortisol), and aging-associated serum markers (VEGF, IGF-1, klotho, and TSLP), and microbiota composition in the complex context involving the COVID-19 pandemic for older adults. Aging is a highly variable process to different tissues, influenced by genes and lifestyle, and the answer to stress is individual and heterogeneous, and dependent on internal and environmental stressors. In addition, the development of new health strategies for the post-COVID-19 period must be considered, since some recovery capacities may have been reduced in older adults, such as neurocognitive, muscular, and immunological functions.

## Limitations

The study design was initially performed to investigate a specific physical activity protocol. However, the lockdown and physical distancing advocated by governmental authorities, made it difficult to find these participants, which justified the low number of participants. It is important to mention that only women were included in T1, since males were a small minority of participants, and they were not comfortable to participate in this new sampled period. Thus, there was more homogeneity for gender-influenced analysis, such as eating habits, inflammation and aging serum markers, and microbiota composition. On the other hand, it is well-document that women take more care of their own health, which could clutter or benefit the present findings. Another point that could be considered is the lack of confirmation about SARS-Cov2 contamination for participants before vaccination, in addition to the lack of information about antibiotic treatment before the pandemic period, conditions that could change the studied parameters. Thus, it must be considered that the present study used self-reported health conditions of participants. However, the findings of this study offer new, potentially useful information for future observations and associations between the health of the older population and the social/physical restrictions imposed by the COVID-19 pandemic.

## Final considerations

There are great concerns regarding the impacts of physical distancing on the health of populations in the whole world. Therefore, due to the greater biopsychosocial vulnerability of older adults, it was hypothesized that this group would undergo physiological changes relevant to their health, besides all of the risks associated to the COVID-19 pandemic. It is important to mention the scarcity of studies focused on the aged population, more specifically in women, which may have been harmful to the discussion of the current data. In addition, the period before physical distancing examined in the present study could also be considered a stressing and anxiety period with modifications for participants, since much information was published about COVID-19, including deaths in Europe, mainly among the older population. In addition, in the same period, no effective treatment or vaccine were available while the virus spread throughout Brazil without effective health control measures implemented in the whole national territory, contributing to insecurity of the participants. The present study showed that 6 months of physical distancing was able to modify health-physical components (BMI, physical activity practice and strength), eating habits and diet quality, serum markers (BDNF, IFN-γ, IL-2, IL-5, and IGF-1), fecal composition (*Blautia* spp.), as well some correlations among these variables. Interestingly, before physical distancing, the more plausible associations were observed among physical parameters, food intake, serum markers, and microbiota composition. However, several associations were lost while others unexpectedly became apparent. The results of the present study suggest negative impacts of physical distancing, such as increased sedentary behavior, BMI, non-protective food consumption and tendency to a more inflammatory profile. However, future studies involving other populations are needed to support interventions that promote the health of older people, both during and after the physical distancing that a pandemic or other events may require.

## Data availability statement

The datasets presented in this study can be found in online repositories. The names of the repository and accession number(s) can be found below: www.ncbi.nlm.nih.gov/bioproject/PRJNA857193.

## Ethics statement

The studies involving human participants were reviewed and approved by the Research Ethics Committee of São Judas Tadeu University. The patients/participants provided their written informed consent to participate in this study. Written informed consent was obtained from the individual(s) for the publication of any potentially identifiable images or data included in this article.

## Author contributions

GL, PL, MB, RA, and SO contributed to the conception and design of the research such as conceptualization, project administration, methodology, resources, and supervision. PL, RA, SO, and MB contributed to the design of the manuscript, wrote and reviewed the manuscript, and contributed to the interpretation and analysis of the data. RO, AG, GM, AB, AL, JA, FM, GL, RAF, PL, and MB contributed to the acquisition of the data. All authors contributed to the article and approved the submitted version.

## Footnotes

1
http://produtos.seade.gov.br/produtos/msp/ren/ren2_001.htm
