# Supplementary material for: Effects of physical distancing by COVID-19 pandemic on diet quality, neurological and immunological markers, and fecal microbiota of Brazilian older women
Source: Front Nutr. 2022 Sep 14;9:972100. doi: 10.3389/fnut.2022.972100 (PMC9534123; doi:10.3389/fnut.2022.972100)
Supplement: Supplementary file 1 [file Data_Sheet_1.docx]

**Table 1:** Spearman correlation between Body mass index, physical activity. and strength and dietary before (T0) and after six months of physical distancing (T1). The R and p values were presented for all analysis and * represents statistical significance level (p<0.05).

|  | T0 | | | | | T1 | | | | | | | | | | | | |
| --- | --- | --- | --- | --- | --- | --- | --- | --- | --- | --- | --- | --- | --- | --- | --- | --- | --- | --- |
|  | **BMI** | **Physical activity** | | | **Strength** |  | | **BMI** | | **Physical activity** | | | | | | **Strength** | | |
|  |  | **Light** | **Moderate** | **Intense** |  |  |  |  |  | **Light** | **Moderate** | | | **Intense** | |  |  |  |
| DST | 0.128  0.645 | 0.168  0.617 | -0.065  0.850 | -0.522  0.118 | 0.208  0.453 |  | -0.158  0.663 | | -0.018  0.984 | | | -0.222  0.643 | -0.356  0.476 | | -0.193  0.545 | |  |  |
| PROTECTIVE  FOODS | 0.037  0.896 | -0.002  0.998 | -0.017  0.962 | -0.52  0.118 | 0.392  0.148 |  | -0.171  0.634 | | -0.073  0.889 | | | -0.482  0.288 | 0  1 | | -0.274  0.384 | |  |  |
| FRUIT AND  VEGETABLES | -0.057  0.838 | -0.103  0.761 | -0.247  0.456 | -0.419  0.218 | **0.517**  **0.050*** |  | -0.488  0.154 | | -0.236  0.600 | | | -0.482  0.288 | 0.231  0.714 | | 0.12  0.708 | |  |  |
| NON-PROTECTIVE | 0.051  0.858 | -0.011  0.984 | 0.083  0.815 | -0.172  0.964 | -0.068  0.810 |  | 0.195  0.586 | | 0.698  0.090 | | | -0.192  0.690 | 0.144  0.964 | | -0.391  0.207 | |  |  |
| PROCESSED  MEAT | 0.22  0.428 | -0.273  0.415 | 0.227  0.499 | 0.082  0.945 | -0.468  0.080 |  | 0.176  0.629 | | -0.079  0.914 | | | 0.32  0.571 | -0.0966  1 | | -0.36  0.248 | |  |  |

**Table 2:** Spearman correlation between Body mass index, physical activity. and strength and serum markers before (T0) and after six months of physical distancing (T1). The R and p values were presented for all analysis and * represents statistical significance level (p<0.05).

|  | T0 | | | | | T1 | | | | | | | | | | | | |
| --- | --- | --- | --- | --- | --- | --- | --- | --- | --- | --- | --- | --- | --- | --- | --- | --- | --- | --- |
|  | **BMI** | **Physical activity** | | | **Strength** |  | | **BMI** | | **Physical activity** | | | | | | **Strength** | |  |
|  |  | **Light** | **Moderate** | **Intense** |  |  |  |  |  | **Light** | **Moderate** | | | **Intense** | |  |  |  |
| BDNF | -0.09  0.758 | 0.078  0.822 | 0.244  0.465 | -0.256  0.473 | 0.156  0.591 |  | 0.629  0.076 | | 0.358  0.522 | | | 0.669  0.233 | 0.664  0.333 | | -0.135  0.71 | |  |  |
| Cortisol | -0.176  0.566 | 0.091  0.804 | 0.039  0.921 | -0.29  0.600 | 0.349  0.241 |  | **0.711**  **0.037*** | | 0.088  0.933 | | | 0.541  0.267 | 0.655  0.333 | | -0.255  0.47 | |  |  |
| Il-2 | 0.275  0.338 | -0.369  0.261 | -0.05  0.883 | 0.46  0.182 | **-0.578**  **0.033*** |  | -0.075  0.854 | | -0.088  0.933 | | | -0.541  0.267 | -0.655  0.333 | | -0.024  0.95 | |  |  |
| IL-5 | 0.031  0.918 | -0.078  0.818 | 0.165  0.621 | 0.419  0.218 | 0.069  0.812 |  | 0.192  0.620 | | 0  1.000 | | | 0.845  0.067 | 0.393  0.667 | | -0.386  0.65 | |  |  |
| IL-6 | 0.062  0.835 | -0.526  0.100 | 0.124  0.714 | -0.094  0.800 | -0.253  0.379 |  | 0.392  0.293 | | 0.224  0.678 | | | -0.514  0.300 | 0.133  1.000 | | 0.279  0.61 | |  |  |
| IL-10 | -0.151  0.602 | -0.069  0.839 | **-0.642**  **0.038*** | -0.547  0.091 | -0.021  0.944 |  | 0.243  0.528 | | 0.265  0.667 | | | -0.439  0.400 | -0.131  1.000 | | 0.193  0.38 | |  |  |
| IFN-γ | -0.018  0.955 | 0.389  0.236 | -0.124  0.714 | -0.27  0.436 | 0.088  0.764 |  | 0.31  0.415 | | -0.177  0.767 | | | 0.541  0.267 | 0.655  0.333 | | 0.181  0.98 | |  |  |
| TNF-α | -0.099  0.735 | -0.531  0.096 | -0.196  0.560 | 0.04  0.927 | -0.192  0.509 |  | -0.084  0.834 | | 0.353  0.522 | | | -0.676  0.200 | -0.131  1.000 | | 0.084  0.91 | |  |  |
| VEGF | -0.213  0.461 | -0.339  0.306 | 0.148  0.663 | 0.013  1.000 | **-0.681**  **0.009*** |  | 0.527  0.150 | | -0.441  0.433 | | | 0.338  0.533 | -0.393  0.667 | | 0.494  0.38 | |  |  |
| IGF-1 | -0.139  0.634 | -0.407  0.213 | **0.793**  **0.006*** | 0.121  0.727 | 0.084  0.776 |  | 0.067  0.868 | | 0.618  0.244 | | | 0.135  0.800 | 0.393  0.667 | | -0.506  0.99 | |  |  |
| Klotho | 0.346  0.224 | **0.789**  **0.006*** | 0.101  0.766 | -0.095  0.809 | -0.051  0.863 |  | -0.109  0.784 | | 0.794  0.100 | | | 0.304  0.600 | 0.655  0.333 | | -0.639  0.59 | |  |  |
| TSLP | -0.141  0.629 | -0.6  0.056 | 0.339  0.304 | 0.324  0.364 | **-0.698**  **0.007*** |  | 0.092  0.819 | | 0.088  0.933 | | | 0.541  0.267 | 0.655  0.333 | | -0.711  0.51 | |  |  |
| IL-6/IL-10 | 0.471  0.091 | 0  1.000 | 0.392  0.232 | 0.539  0.091 | -0.271  0.346 |  | 0.025  0.958 | | 0  1.000 | | | -0.034  1.000 | 0.131  1.000 | | 0.133  0.39 | |  |  |
| TNF-α/IL-10 | 0.24  0.406 | -0.252  0.452 | 0.382  0.244 | 0.607  0.055 | **-0.599**  **0.026*** |  | -0.285  0.454 | | 0  1.000 | | | -0.676  0.200 | -0.131  1.000 | | -0.217  0.31 | |  |  |
| IFN-γ/IL-5 | -0.11  0.707 | 0.178  0.597 | -0.201  0.549 | -0.418  0.218 | 0.035  0.906 |  | 0  1.000 | | 0  1.000 | | | -0.507  0.333 | 0.131  1.000 | | 0.374  0.89 | |  |  |

**Table 3:** Spearman correlation between Body mass index, physical activity. and strength and fecal microbiota before (T0) and after six months of physical distancing (T1). The R and p values were presented for all analysis and * represents statistical significance level (p<0.05).

|  | T0 | | | | | T1 | | | | | | | | | | | |
| --- | --- | --- | --- | --- | --- | --- | --- | --- | --- | --- | --- | --- | --- | --- | --- | --- | --- |
|  | **BMI** | **Physical activity** | | | **Strength** |  | | **BMI** | | **Physical activity** | | | | | | **Strength** | |
|  |  | **Light** | **Moderate** | **Intense** |  |  |  |  |  | **Light** | **Moderate** | | | **Intense** | |  |  |
| *Akkermansia* spp. | -0.038  0.895 | -0.233  0.487 | -0.22  0.512 | 0.189  0.600 | **0.529**  **0.045*** |  | 0.427  0.218 | | 0.128  0.757 | | | -0.447  0.264 | 0.024  0.964 | | -0.523  0.084 | |  |
| *Blautia* spp. | 0.195  0.483 | -0.021  0.954 | 0.065  0.848 | 0.284  0.418 | **0.517**  **0.05*** |  | 0.456  0.186 | | 0.182  0.668 | | | 0.241  0.561 | 0.312  0.464 | | -0.063  0.847 | |  |
| *Bifidocabterium spp.* | -0.043  0.879 | -0.114  0.737 | -0.11  0.748 | 0.58  0.073 | 0.003  0.994 |  | 0.193  0.589 | | 0.494  0.213 | | | -0.211  0.608 | 0.455  0.286 | | -0.449  0.143 | |  |
| *Eubacterium spp.* | -0.191  0.492 | -0.252  0.452 | -0.057  0.869 | -0.108  0.764 | 0.389  0.152 |  | 0.128  0.725 | | 0.194  0.644 | | | -0.241  0.561 | -0.171  0.714 | | -0.053  0.873 | |  |
| *Faecalibacterium spp.* | **-0.55**  **0.036*** | **-0.627**  **0.043*** | 0.54  0.090 | 0.283  0.400 | 0.181  0.516 |  | 0.219  0.542 | | -0.012  0.988 | | | 0.723  0.056 | -0.062  0.893 | | -0.361  0.247 | |  |
| *Lactobacillus spp.* | -0.075  0.789 | -0.52  0.103 | 0.415  0.202 | 0.482  0.091 | -0.211  0.445 |  | 0.141  0.693 | | -0.104  0.804 | | | -0.096  0.805 | -0.536  0.196 | | -0.332  0.287 | |  |
| *Roseburia spp.* | **-0.618**  **0.016*** | -0.252  0.452 | -0.22  0.512 | -0.31  0.382 | 0.258  0.351 |  | 0.565  0.093 | | 0.267  0.519 | | | 0.342  0.404 | 0.265  0.536 | | -0.14  0.662 | |  |
| Bacteroidetes | 0.318  0.246 | 0.124  0.713 | -0.065  0.851 | 0.528  0.109 | **-0.59**  **0.023*** |  | -0.128  0.723 | | 0.339  0.405 | | | -0.165  0.695 | 0.016  1.000 | | -0.086  0.788 | |  |
| Firmicutes | -0.029  0.920 | -0.037  0.918 | -0.296  0.371 | -0.337  0.345 | **0.587**  **0.024*** |  | 0.462  0.181 | | -0.352  0.389 | | | 0.342  0.404 | -0.343  0.393 | | -0.425  0.169 | |  |
| Firmicutes/Bacteroidetes | -0.064  0.082 | -0.188  0.578 | -0.053  0.882 | -0.256  0.473 | 0.118  0.673 |  | -0.341  0.330 | | 0.497  0.214 | | | **-0.761**  **0.04*** | 0.016  1.000 | | -0.033  0.919 | |  |
| Lactobacillaceae | -0.382  0.159 | 0.056  0.872 | 0.209  0.530 | -0.33  0.382 | 0.461  0.085 |  | -0.204  0.566 | | -0.088  0.834 | | | -0.15  0.721 | -0.49  0.250 | | 0.062  0.847 | |  |
| Lachnospiraceae | **-0.559**  **0.032*** | -0.394  0.230 | -0.062  0.530 | 0.013  1.000 | 0.075  0.790 |  | 0.073  0.845 | | 0.121  0.776 | | | -0.609  0.118 | 0.031  0.964 | | -0.028  0.934 | |  |

**Table 4:** Spearman correlation between dietary and neurological. immunological. and aging-associated markers before (T0) and after six months of physical distancing (T1). The p and R values were presented for all analysis and * represents statistical significance level (p<0.05).

|  | **T0** | | | | | | | | | | | | | | **T1** | | | | | | | | | | | | | |
| --- | --- | --- | --- | --- | --- | --- | --- | --- | --- | --- | --- | --- | --- | --- | --- | --- | --- | --- | --- | --- | --- | --- | --- | --- | --- | --- | --- | --- |
|  | **DST** | | | **PROTECTIVE**  **FOODS** | | | **FRUIT AND**  **VEGETABLES** | | | **NON-PROTECTIVE** | **PROCESSED**  **MEAT** | | | | **DST** | | | **PROTECTIVE**  **FOODS** | **FRUIT AND**  **VEGETABLES** | | | | **NON**  **PROTECTIVE** | | | **PROCESSED**  **MEAT** | | |
| **BDNF** | **0.550**  **0.043*** | | 0.500  0.068 | | | 0.377  0.183 | | | 0.337  0.240 | | | 0.203  0.485 | | **-0.750**  **0.016*** | | | -0.630  0.057 | | | **-0.660**  **0.042*** | | -0.202  0.570 | | | - 0.137  0.716 | | |  |
| **CORTISOL** | 0.496  0.087 | | 0.556  0.051 | | | 0.494  0.081 | | | 0.317  0.291 | | | 0.354  0.234 | | 0.062  1.000 | | | 0.430  0.218 | | | **-0.723**  **0.022*** | | 0.292  0.411 | | | 0.272  0.464 | | |  |
| **IL 2** | -0.037  0.901 | | - 0.167  0.570 | | | -0.361  0.203 | | | 0.007  0.984 | | | 0.181  0.534 | | 0.612  0.067 | | | 0.455  0.190 | | | -0.182  0.614 | | 0.468  0.174 | | | 0.545  0.119 | | |  |
| **IL 5** | 0.175  0.547 | | 0.004  0.89 | | | -0.097  0.739 | | | 0.128  0.664 | | | -0.53  0.854 | | 0.503  0.144 | | | 0.060  0.860 | | | -0.328  0.353 | | 0.340  0.334 | | | 0.506  0.149 | | |  |
| **IL 6** | -0.073  0.843 | | -0.091  0.804 | | | 0.171  0.636 | | | - 0.052  0.896 | | | 0.270  0.480 | | -0.433  0.212 | | | -0.238  0.505 | | | -0.321  0.361 | | 0.263  0.457 | | | -0.548  0.109 | | |  |
| **IL 10** | -0.067  0.857 | | -0.152  0.674 | | | 0.244  0.494 | | | -0.088  0.812 | | | **-0.674**  **0.046*** | | -0.042  0.918 | | | 0.006  1.000 | | | -0.219  0.542 | | 0.103  0.777 | | | - 0.078  0.872 | | |  |
| **IFN-𝛾** | **-0.732**  **0.020*** | | -0.559  0.0975 | | | 0.000  1.000 | | | **-0.652**  **0.045** | | | -0.270  0.480 | | -0.370  0.296 | | | -0.624  0.060 | | | -0.109  0.766 | | -0.322  -0.361 | | | -0.350  0.337 | | |  |
| **TFN-α** | -0.037  0.924 | | -0.079  0.830 | | | 0.341  0.331 | | | -0.119  0.744 | | | -0.180  0.654 | | -0.127  0.733 | | | -0.406  0.247 | | | -0.419  0.227 | | 0.328  0.353 | | | -0.195  0.612 | | |  |
| **VEGF** | 0.390  0.264 | | -0.413  0.235 | | | **-0.652**  **0.046*** | | | -0.088  0.812 | | | **0.809**  **0.009*** | | -0.091  0.811 | | | -0.042  0.918 | | | -0.164  0.649 | | -0.146  0.688 | | | 0.234  0.542 | | |  |
| **IGF-1** | 0.098  0.720 | | 0.255  0.474 | | | 0.189  0.599 | | | -0.157  0.665 | | | 0.225  0.568 | | 0.127  0.733 | | | -0.006  1.000 | | | -0.571  0.089 | | **0.851**  **0.003*** | | | 0.078  0.872 | | |  |
| **KLOTHO** | -0.037  0.924 | | 0.000  1.000 | | | 0.018  0.966 | | | -0.326  0.358 | | | 0.090  0.849 | | -0.333  0.349 | | | -0.067  0.865 | | | -0.219  0.542 | | 0.176  0.624 | | | -0.156  0.702 | | |  |
| **TSLP** | -0.104  0.777 | | -0.328  0.353 | | | -0.323  0.360 | | | 0.188  0.603 | | | 0.225  0.568 | | 0.055  0.892 | | | -0.406  0.247 | | | -0.480  0.162 | | 0.243  0.493 | | | 0.000  100 | | |  |
| **IL 6/IL 10** | -0.274  0.440 | -0.255  0.474 | | | -0.445  0.198 | | | 0.025  0.952 | | | | **0.809**  **0.009** | -0.188  0.607 | | | -0.067  0.865 | | | | -0.970  0.792 | 0.298  0.400 | | | -0.545  0.119 | | |  |  |
| **TNF α /IL 10** | -0.201  0.575 | | -0.176  0.624 | | | -0.317  0.369 | | | -0.113  0.758 | | | **0.719**  **0.029*** | | -0.127  0.733 | | | -0.430  0.218 | | | -0.304  0.391 | | 0.255  0.475 | | | -0.271  0.464 | | |  |
| **IFN 𝛾 /IL 5** | -0.506  0.139 | -0.286  0.402 | | | 0.128  0.725 | | | **-0.778**  **0.010*** | | | | -0.405  0.268 | -0.624  0.060 | | | 0.248  0.492 | | | | 0.176  0.624 | -0.347  0.329 | | | **-0.740**  **0.025*** | | |  |  |
